# The Efficacy of Opioid-Free General Anesthesia in the Management of Hip Surgeries in Elderly Patients

**DOI:** 10.7759/cureus.11295

**Published:** 2020-11-02

**Authors:** Georges R Assaf, Fares Yared, Christelle Abou Boutros, Deoda Maassarani, Racha Seblani, Clara Khalaf, Jean El Kaady

**Affiliations:** 1 Anesthesiology, Lebanese Hospital Geitaoui-University Medical Center, Beirut, LBN; 2 Internal Medicine, Lebanese Hospital Geitaoui, Beirut, LBN

**Keywords:** non-opioid free general anesthesia, time to extubate, postoperative morphine use, opioid free general anesthesia, morphine use

## Abstract

Introduction

Perioperative management of elderly patients differ from young patients due to physiologic and pharmacologic differences related to aging. Moreover, assessment for perioperative parameters and risks between age-matched elderly patients should be discerned while planning for the anaesthesia regimen. The latter could consist of opioid-free general anaesthesia (OFA) or non-opioid-free general anaesthesia (NOFA). Among the parameters for assessing the regimen’s efficacy, time to extubate and pain control should be included. However, it is not yet established whether OFA could replace NOFA as a standard regimen for management of hip fracture. Therefore, the aim of this study is to evaluate the efficacy of OFA for hip surgeries in elderly patients.

Methods

This is a retrospective study consisting of patients undergoing hip surgeries under opioid-free or opioid-induced general anaesthesia. Two groups were defined: Group 1 consisting of treated patients using OFA and Group 2 consisting of treated patients using NOFA. Patient demographics (age, sex, and weight), mean time to extubate and mean dose of morphine after recovery were computed. Postoperative morphine use was assessed for up to 24 hours. Comparison of the computed data was conducted between both groups. Mean postoperative morphine use was compared using the Mann-Whitney U-test. The remainder of the means were compared using independent t-test. Qualitative data were compared using Fisher’s exact test. Level of significance was set at p<0.05.

Results

A total of 73 patients were included. Group 1 consisted of 37 patients (12 were males with mean age 77±14 years) who underwent hip fracture procedure whereas Group 2 consisted of 36 patients (18 were males with mean age 73±17). There were significant differences when comparing sex, weight, and time to extubate (6.8±3 and 10±5 minutes in Groups 1 and 2, respectively; p<0.05). There were six patients in Group 1 and 17 patients in Group 2 that required postoperative morphine administration. Mann-Whitney U comparison of postoperative morphine use yielded significant differences (4.8±3 and 14.65±13 mg in Groups 1 and 2, respectively; p=0.001).

Discussion

This is the first study that assessed the efficacy of OFA compared to NOFA in the management of hip fractures. Non-significant differences in age might suggest that both groups are age matched. In addition, significant differences in time to extubate might help in reducing impact on ventilation, maintaining safe anaesthesia while minimizing intraoperative work overflow. Patients in Group 1 required less morphine in the postoperative setting than in Group 2. This might be explained by the sensation of paraesthesia which might have been confused with pain.

Conclusions

OFA could be considered in hip management in elderly patients; femoral and lateral cutaneous block seemed to act as morphine sparing in operative and postoperative settings by providing significantly less time to extubate with less postoperative morphine requirement.

## Introduction

The perioperative management of an elderly patient is significantly different from that of a young patient due to physiological and pharmacological changes associated with advanced age [1].

Furthermore, overall profiles, risk assessments and perioperative needs should be distinguished as they are different even between age-matched elderly patients [2]. Pre-operative morphine use as well as postoperative use for pain control increase time to extubate and reduce post-operative recovery in elderly patients [1,3].

Hip fractures are serious injuries in the elderly category which could be life-threatening especially if associated with comorbidities [4,5].

General anesthesia or non-opioid-free anesthesia (NOFA) with the regular use of narcotics, hypnotics and muscle relaxants reduces ventilatory response in elderlies to hypoxemia and hypercapnia is the gold standard [6].

Opioid-free general anesthesia (OFA) was introduced to avoid tolerance and hyperalgesia, allowing a reduction in postoperative opioids. OFA focused initially on postoperative respiratory safety for patients undergoing ambulatory surgery and for obstructive sleep apnea syndrome [7].

However, it is not well established in hip fracture management whether OFA could replace a NOFA regimen.

Therefore, the aim of this study is to evaluate the efficacy of OFA in the management of hip fracture in comparison to NOFA.

## Materials and methods

Patients selection

This is an IRB approved retrospective study that was conducted in the Anesthesiology Department of the Lebanese Hospital Geitaoui UMC. Between November 2018 and June 2020. Data from admitted patients for hip surgeries under general anesthesia were considered for the study. Two groups were established: Group 1 consisted of patients undergoing hip surgery under OFA whereas Group 2 consisted of patients undergoing hip surgery under NOFA.

Demographic parameters (age, sex and weight), side of surgery, procedure time, time to extubate as well as dose of post-operative morphine use during the first 24 hours were computed. Only total hip Replacement procedure was included in this study. Patients of interest were those who received general anesthesia under OFA and NOFA

Patients were excluded if they had underlying conditions such as Alzheimer’s Disease, mental retardation, renal failure and failure of loco regional anesthesia. Patients with incomplete medical records or follow up and complications in the postoperative setting were also excluded.

Study protocol

The patients were all admitted to operating room monitored after undergoing a pre-operative checklist. The induction was started after preoxygenation. All patients received sufentanil at a dose of 0.1-0.2 µg/kg followed by xylocaine 1mg/kg, propofol 2mg/kg and rocuronium 1mg/kg.

After ventilation for three minutes, they were all intubated and ventilated after the checkup of the endotracheal tube. OFA patients received femoral nerve block and lateral cutaneous nerve block using a hyperechogenic needle 50 mm length, all of these patients received 75 mg of ropivacaine diluted in 20 ml normal saline. The volume of injection was 15 ml on the femoral nerve and 5 ml on the lateral cutaneous nerve. Ten minutes later, patients were positioned on the orthopedic table, the operation started 30 minutes later after surgical installation and field preparation.

NOFA patients who did not receive any bloc were positioned, and the surgery started after 30 minutes of the time to prepare the surgical field.

At the end of surgery, propofol was stopped at skin closure and the muscle relaxant antagonist was delivered after the skin dressing was finished. The time to extubate was counted starting the dressing time till the extubating was practiced.

All patients in both groups received sufentanil during operation when a 20% increase in the blood pressure or heart rate was noticed.

The postoperative data were collected from the nurse sheet over 24 hours and both groups benefited from paracetamol 1g intravenous systematically with morphine IM 0.1mg/kg every six hours on demand of the patients.

In the postoperative prescription, intra-muscular morphine was given only to patients with pain scale VAS (visual analogue scale) superior or equal to 4/10.

The nerve block was done by an experienced anesthesiologist; first step was proper disinfection followed by a complete ultrasound-guided needle insertion using the ”in plain technique”.

Statistical analysis

Qualitative data were compared between both groups using Fisher’s exact test (sex comparison). The remaining demographic parameters and means of procedure time, time to extubate were compared using the independent t-test. Means of postoperative morphine use were compared between Group 1 and Group 2 using Mann-Whitney U-test. Level of statistical significance for comparison between groups was set at p<0.05.

## Results

A total of 73 patients were included (among which there were 43 females; total mean age of 75±16 years). Group 1 consisted of 37 patients receiving OFA (among which there were 25 females; mean age of 77±17 years). Mean time to extubate was 6.8±3 minutes [8]. Group 2 consisted of 36 patients among which 18 were females. Mean age was 73±17 years and mean time to extubate was 10.1±5 minutes. Remaining demographic parameters and means are displayed in Table 1.

**Table 1:** Patient demographics and calculated means NOFA: non-opioid-free general anesthesia; OFA: opioid-free general anesthesia.

	Number of Subjects	Sex		Age (years)	Weight (kg)	Procedure		Procedure Time (minutes)	Time to Extubate (Minutes)
		M	F	Mean ±SD	Mean ± SD	Left Hip Fracture	Right Hip Fracture	Mean ± SD	Mean ± SD
NOFA	36	18	18	73±17	71±12	14	22	146±41	10.1±5
OFA	37	12	25	77±14	65±13	21	16	121±53	6.8±3
Total	73	30	43	75±16	68±13	35	38	133±48	8.5±4

When comparing demographic parameters between both groups, significant differences were found in sex and weight (p-values were <0.01 and 0.04, respectively). There were no significant differences when comparing age between both groups (p-value = 0.5).

In addition, significant differences were found when comparing time to extubate between both groups (p-value = 0.001).

**Figure 1:**
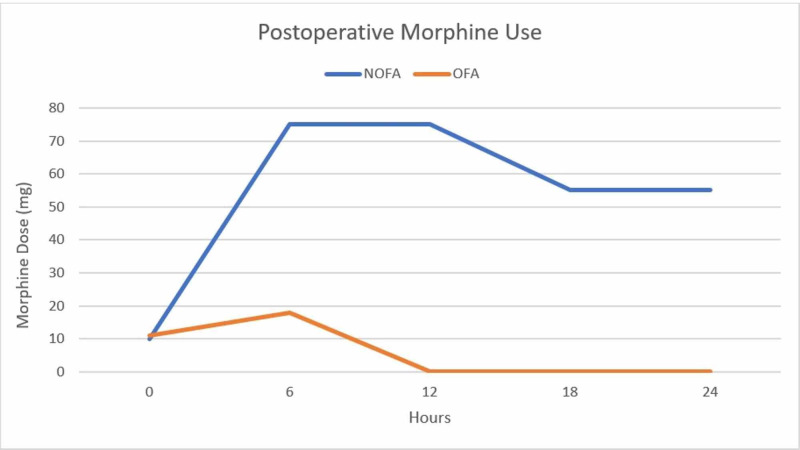
Postoperative morphine use in OFA and NOFA patients during the 24-hour follow-up NOFA: non-opioid-free general anesthesia; OFA: opioid-free general anesthesia.

There were six patients in Group 1 that required postoperative morphine use (16.6% of Group 1) with a mean dose of 4.8±3 mg. There were 17 patients in Group 2 that required postoperative morphine use (46% of Group 2) with a mean dose of 14.65±13 mg. Morphine use in the 24-hour postoperative setting was summarized in Figure 1 and Table 2.

**Table 2:** Postoperative morphine use and comparison between patients receiving OFA and NOFA NOFA: non-opioid-free general anesthesia; OFA: opioid-free general anesthesia.

Group	Number of Subjects	Proportion (%)	Mean ± SD (mg)	Mann-Whitney U-Test
NOFA	17	17/37(46%)	14.65±13	p=0.001
OFA	6	6/36(16.6%)	4.8±3	

Mann-Whitney U-test yielded significant differences when comparing mean postoperative morphine use between Groups 1 and 2 (p-value = 0.001).

## Discussion

This is the first study that aimed to evaluate the efficacy of OFA in hip management in elderly patients. Comparisons of both groups yielded a non-significant difference in age which might suggest that the groups are age matched. Significant differences were obtained in weight comparison between both groups. Group 2 had a significantly increased weight distribution than Group 1.

When comparing means related to the procedure, significant differences were found when assessing mean time to extubate.

Group 1 had a significantly less time to extubate (6.8±3 minutes) than Group 2 (10.1±5 minutes; p-value=0.001) which can influence the workflow of the operating room. This significant difference might suggest that the OFA regimen provides less ventilatory impact on patients while maintaining adequate and safe anesthesia. However, a study conducted by Bhardwaj et al [8] comparing outcomes between OFA and NOFA in obese patients undergoing urological procedures showed a significantly greater time to extubate in patients receiving OFA. These conflicting results might be explained by the procedure type. In addition, OFA patients in the aforementioned study had a mean weight inferior to NOFA patients. This finding is concordant with the current study.

In the postoperative setting, patients in Group 1 required less morphine (mean morphine dose 4.8±3 mg) than those in Group 2 (mean morphine dose 14.65±13 mg); Mann-Whitney U-test was conducted to compare both groups by taking into account the comparison between the low number of patients that required morphine use. Only four patients from Group 1 requested in the direct postoperative setting. This could be related to the sensation of paresthesia in the lower limb which may be confused with pain. The remaining two patients received morphine after 18 hours.

Seventeen patients from Group 2 received morphine among which most patients required it in the first six to 12 hours in the postoperative setting. Less patients requested morphine 18 hours after the end of the procedure.

Moreover, OFA patients from Bhardwaj’s comparative study had required less morphine in the postoperative setting compared to NOFA patients [8]. This finding is also concordant with the current study.

Limitations

This study is a retrospective comparison of patients receiving two different regimens of anesthesia. A proper bias control related to subjective assessment of pain is a limitation as well in the assessment of morphine administration in the postoperative setting. In addition, postoperative assessment between both groups regarding adverse effects related to anesthesia could not have been obtained.

Future studies should establish a randomized controlled trial between OFA and NOFA in order to address potential differences between the efficacy and safety of these different regimens.

## Conclusions

Despite conducting a retrospective study with a reduced number of subjects, outcomes from these comparisons might reveal interesting results. Between-group comparisons showed that time to extubate was significantly lower when administering OFA than NOFA. In addition, postoperative patients under OFA required less morphine administration than postoperative patients under NOFA. Future studies should conduct randomized controlled trials on larger samples to properly assess the efficacy of OFA.
